# Genome-wide association study of growth, carcass, and meat quality traits in Liangshan Yanying chickens

**DOI:** 10.1016/j.psj.2026.107768

**Published:** 2026-09-12

**Authors:** Yuhang He, Qiang Wang, Jinghe Li, Ming Yang, Jun Zou, Yuqi Chen, Jianian Zhao, Jianxing Jiang, Ruxue Liu, Xinyu Wei, Xiaochan Yuan, Zhaoyou Zheng, Xiyu Tan, Qiao Xiang, Jianfeng Huang, Renbo Tong, Hehe Liu, Anqi Huang

**Affiliations:** a College of Life Sciences, Sichuan Agricultural University, Ya'an, Sichuan, 625014, China; b Liangshan Yi Autonomous Prefecture Academy of Agricultural Sciences, Xichang, Sichuan, 615000, China; c State Key Laboratory of Swine and Poultry Breeding Industry, College of Animal Science and Technology, Sichuan Agricultural University, Chengdu, Sichuan, 611130, PR China

**Keywords:** Liangshan Yanying chicken, Genome-wide association study, Meat quality trait, Growth trait, Carcass trait

## Abstract

This study investigated the genetic basis of growth, carcass, and meat quality traits in Yanying chickens, an indigenous breed adapted to high-altitude environments. A total of 19 growth traits and 29 carcass and meat quality traits were measured, and **genome-wide association study (GWAS)** was performed to identify loci associated with these economically important traits. In total, 455 significant **single nucleotide polymorphisms (SNPs)** were identified, and 176 candidate genes were obtained. Among these, 1 had been directly associated with the corresponding trait in previous chicken studies, while approximately 5 others had been implicated in related traits. Further regional association and **linkage disequilibrium (LD)** analyses highlighted *RYR2* and *ARNTL* as positional candidate genes associated with spleen weight and breast muscle pH, respectively. In addition, expression-based analysis provided additional evidence consistent with *ARNTL* being a positional candidate gene for breast muscle pH. Functional enrichment analysis identified significant **Gene Ontology (GO)** terms related to cell adhesion and calcium ion binding, whereas **Kyoto Encyclopedia of Genes and Genomes (KEGG)** analysis suggested nominal enrichment of ECM-receptor interaction and MAPK signaling pathways. Overall, these findings provide new insight into the genetic architecture of growth, carcass, and meat quality traits in this indigenous breed and offer useful information for future functional validation and genetic improvement of local poultry populations.

## Introduction

Chicken is an important source of animal protein worldwide because of its high nutritional value and wide consumer acceptance. In recent years, consumer demand has expanded beyond rapid growth and high yield to include better flavor, texture, and nutritional quality. As a result, indigenous chicken breeds have attracted increasing attention in breeding programs because they often exhibit desirable meat quality, distinctive flavor, and good adaptation to local environments.

The Yanying chicken is an indigenous breed native to the Liangshan region of Sichuan, China, and was included in the China National Livestock and Poultry Genetic Resources Committee Office (2025). This breed has long been raised at elevations of 1,800 to 2,800 m under cold and hypoxic conditions and has developed distinctive adaptation to high altitude, together with desirable meat quality and strong stress resistance (Yu et al., 2026). Previous studies have shown that chickens adapted to high altitude may exhibit enhanced antioxidant capacity, adaptive immune regulation, and altered lipid metabolism under hypoxic conditions (Feng et al., 2023; Cao et al., 2024; Hao et al., 2025), which may contribute to survival and production performance in extreme environments (Wang et al., 2015; Du et al., 2022). However, the genetic basis underlying economic traits such as growth and meat quality in Yanying chickens remains unclear, and the regulatory mechanisms of these traits await systematic elucidation.

Growth, carcass, and meat quality traits are major economic traits in poultry because they directly affect breeding efficiency and product value. Previous studies on these traits have mainly been conducted in commercial chickens and common indigenous breeds. For example, in Wenshang Barred chickens, *GH, IGF1*, and *IGF2* were significantly associated with body measurement traits, including keel length, breast width, and breast depth (Yang et al., 2022). In Beijing-You and Silkie chickens, polymorphisms in *IGF1* and *IGF2* were also associated with body weight, shank length, shank circumference, and some carcass traits (Tang et al., 2010). Together, these findings support an important role of the GH-IGF axis in chicken growth and body size development. Furthermore, large scale genomic analyses in Chinese indigenous chickens identified *LCORL, LDB2*, and *PPARGC1A* as important candidate genes for body weight and body size (Wang et al., 2024). GWAS has also identified genes associated with carcass and meat quality traits. In an F2 population derived from Luxi Game chickens and recessive white broilers, *FAM184B, QDPR*, and *LDB2* were significantly associated with multiple slaughter traits (Liu et al., 2021). In addition, genes such as *PPP1CC* (Sun et al., 2024) and *BCMO1* (Le Bihan-Duval et al., 2011) have also been implicated in meat color variation. Overall, these studies indicate that chicken economic traits are complex quantitative traits controlled by multiple genes and provide a useful basis for marker-assisted selection and genetic improvement. In contrast, studies in high altitude chickens have mainly focused on hypoxia adaptation, and evidence on economic traits remains limited. For example, in Tibetan chickens, *MyoD* and *Myf5* have been associated with growth traits (Zhang et al., 2018b), whereas *SFRP5* (Li et al., 2016) and *FGF1/FGF10* (Zhang et al., 2018a) have been linked to intramuscular fat deposition. However, studies on other economic traits, particularly systematic GWAS of growth, carcass, and meat quality traits, remain scarce in high altitude chickens.

In this study, GWAS was performed to identify genomic loci associated with growth, carcass, and meat quality traits in Yanying chickens. To further refine the GWAS signals, **linkage disequilibrium (LD)** analysis, LocusZoom analysis, and gene annotation were performed to identify candidate regions and genes associated with breast muscle pH and spleen weight. This study provides new insight into the genetic architecture of growth, carcass, and meat quality traits in Yanying chickens under high-altitude conditions and may support future genetic improvement of indigenous poultry breeds.

## Materials and methods

### Ethics statement

All animal procedures involving chickens were conducted in accordance with a protocol approved by the **Animal Ethics and Welfare Committee (AEWC)** of Sichuan Agricultural University, China.

### Measurement of growth traits and blood sample collection

Yanying chickens were used as the experimental population in this study, and growth traits were measured in selected individuals at different developmental stages. The birds were raised under uniform management at the farm of Xichang Tongxin Agriculture and Animal Husbandry Technology Co., Ltd. Each bird was fitted with a QR code wing tag for individual identification. All birds were reared on the same farm throughout the experimental period. They were housed in stacked cages from 0 to 4 wk of age and on raised net flooring from 5 to 20 wk of age. Throughout the experiment, all birds were maintained under the same stocking density and rearing conditions, with free access to feed. Body weight and body measurement data were collected at 0, 2, 4, 6, 8, and 12 wk of age. Body measurements included body slope length, keel length, chest width, chest depth, shank length, shank girth, and pelvic width. Body weight and body measurements were recorded in accordance with the *Terminology and Measurement Methods for Poultry Production Performance [NY/T 823]*. All measurements were performed by the same trained team using standardized procedures and calibrated instruments. The effective sample size for growth traits varied across measurement stages because phenotype records were unavailable for some individuals at specific time points.

At 12 wk of age, blood samples were collected from the wing vein of all 200 Yanying chickens, transferred into anticoagulant tubes, and stored at −20°C for subsequent whole genome sequencing.

### Measurement of carcass and meat quality traits

At 12 wk of age, 200 Yanying chickens were subjected to carcass and meat quality measurements after 12 h of feed withdrawal, with free access to water. Live body weight was recorded before slaughter. The chickens were then slaughtered by exsanguination through severing of the carotid artery, followed by defeathering and evisceration. The following carcass traits were recorded: dressed weight, bilateral breast muscle weight, heart weight, gizzard weight, glandular stomach weight, spleen weight, liver weight, lung weight, bilateral leg muscle weight, skin fat weight, bilateral foot weight, head weight, giblet weight, half-eviscerated weight with giblets, and eviscerated weight. Left breast muscle thickness was measured at the same time. Breast muscle yield, leg muscle yield and half-eviscerated yield with giblets were then calculated based on these measurements.

Meat color (L^∗^,a^∗^,b^∗^), pH, shear force, and **intramuscular fat (IMF)** content of the left breast muscle were measured according to the methods described by Yang et al. (2026). In addition, moisture, fat, and protein contents of the breast muscle were determined using a near infrared spectrometer (Xingchen F4020, Guangdong Spectrastar Instruments Co., Ltd., Guangzhou, China).

Due to missing phenotype records and sample loss during collection or processing, the actual effective sample sizes for carcass and meat quality traits were all less than the initial 200 chickens.

### Whole-genome resequencing and SNP calling

Whole-genome resequencing was performed as previously described by Yu et al. (2026), with the same experimental and analytical procedures unless otherwise specified. Briefly, genomic DNA was extracted from blood samples collected from 200 Yanying chickens. DNA quality and purity were assessed by agarose gel electrophoresis and NanoDrop spectrophotometry. Paired end libraries were constructed following the standard Illumina HiSeq X Ten protocol and sequenced at Bio-company (Berry Genomics, Beijing, China), generating 150 bp paired end reads with an average sequencing depth of approximately 10 × per individual. Clean reads were aligned to the chicken reference genome bGalGal1.mat.broiler.GRCg7b (GCF_016699485.2) using BWA aln. **Single nucleotide polymorphisms (SNP)** calling was performed using GATK HaplotypeCaller to generate gVCF files. The resulting SNP set was further filtered in PLINK (version 1.90) using the following criteria: minor allele frequency (MAF) > 0.05, major allele frequency < 0.99, missing genotype rate < 0.1, and retention of biallelic SNPs only.

### Genome-wide association study

Genome-wide association analysis was conducted using the mixed linear model implemented in EMMAX, as described by Yu et al. (2026). The model was defined as:

y=Xα+Zβ+Wμ+e

where y is the vector of phenotypic observations; Xα represents fixed effects, with sex included as a covariate; Zβ represents the effect of the tested SNP, with β denoting the allele substitution effect; Wμ denotes the random polygenic effect of individuals, whose variance-covariance structure was modeled using the genomic relationship matrix to account for relatedness among individuals; e is the residual error. The genomic relationship matrix was constructed in EMMAX using genome-wide SNPs retained after PLINK quality control, with genotype standardization followed by IBS-based kinship matrix calculation. Manhattan plots and quantile-quantile (Q-Q) plots were generated using the qqman package in R (version 3.5.1). Bonferroni-corrected thresholds were calculated as *P* = 0.01/N (-log_10_*P* = 9.07) and *P* = 0.05/N (-log_10_*P* = 8.38), where N is the total number of SNPs (11,859,518). The gray line in the Manhattan plots indicates the more stringent threshold of 9.07 used for subsequent analyses, while SNPs with -log_10_*P* ≥ 8.38 can be considered genome-wide significant. Separately, population structure was evaluated by **principal component analysis (PCA)** using PLINK (version 1.90) based on genome-wide SNPs retained after quality control. PCA was used only for population-structure assessment, and the plot was generated in R (version 3.5.1).

### Linkage disequilibrium analysis

After GWAS, regional association analyses were performed for the top SNPs of traits showing clear regional clustering of associated SNPs. For each top SNP, a ± 1 Mb flanking region was used for LocusZoom visualization, and pairwise LD between the top SNP and surrounding SNPs was calculated using PLINK v1.90. Regional association plots were generated using R v3.5.1. SNPs (r^2^ > 0.4) with the top SNP were retained for subsequent LD block analysis, and LD blocks were defined using Haploview with the solid spine of LD method.

### Gene annotation and enrichment analysis

Based on the chicken reference genome (GCF_016699485.2) and annotation files, this study employed the SnpEff software (Cingolani et al., 2012) to perform gene annotation on significant SNPs (-log_10_*P* ≥ 9.07), thereby screening candidate genes within and surrounding these significant SNPs. KOBAS (version 3.0, http://bioinfo.org/kobas) was used for **Kyoto Encyclopedia of Genes and Genomes (KEGG)** pathway enrichment analysis and **Gene Ontology (GO)** function enrichment analysis. Both raw *P* values and adjusted *P* values were reported. GO terms and KEGG pathways with adjusted *P* < 0.05 were considered significantly enriched, whereas terms or pathways with raw *P* < 0.05 but adjusted *P* ≥ 0.05 were considered nominally enriched and interpreted as suggestive results.

### Transcriptome-wide expression-trait association analysis

To provide additional expression-level support for candidate genes identified in the GWAS, we performed a transcriptome-wide expression-trait association analysis using unpublished pectoralis major RNA-seq data from the same cohort of Yanying chickens, a dataset originally generated within our research group. The association between gene expression and the quantitative trait was modeled using a multiple linear regression framework with covariate adjustment. TPM expression values were log₂-transformed and normalized, and the phenotypic data were processed using the same procedure. PCA was applied to the expression matrix to capture major expression structure and to control for population stratification, sampling batch, and technical variation; sex was also included as a fixed covariate. For each gene, regression coefficients and *P*-values were estimated to identify significant associations. Manhattan plots were generated using CMplot package in R (version 3.5.1), with the Bonferroni-corrected significance threshold set at 0.05 divided by the total number of tested genes, and the target candidate genes were highlighted.

## Results

### Phenotypic statistics

Phenotypic statistics were summarized for growth, carcass, and meat quality traits in Yanying chickens. For growth traits (Table 1), body weight and body measurements showed moderate phenotypic variation across age groups. The **coefficient of variation (CV)** ranged from 6.92% to 17.05%, with the highest CV observed for body weight at 4 wk (17.05%). The standard deviations and medians also indicated noticeable individual variation among growth traits. Most growth traits were continuously distributed and approximately normal (Supplementary Fig. S1), which is consistent with the characteristics of quantitative traits. For carcass and meat quality traits, 29 phenotypes were recorded, including 19 carcass traits and 10 meat quality traits. Overall, these traits showed relatively large variation (Table 2, Table 3). In particular, spleen weight, a*, b*, shear force, fat content and the intramuscular fat contents of fresh and dried meat all had CV values greater than 30%, indicating substantial dispersion among individuals. Most carcass and meat quality traits were continuously distributed (Supplementary Fig. S2). Most followed an approximately normal distribution, whereas a few showed skewed distributions.

**Table 1:** Statistical result of growth traits in Yanying chickens.

Age (week)	Traits	N	Mean	SD	Median (P25, P75)	Min	Max	CV (%)
0	birth weight (g)	149	34.6	3.42	34.2 (32.4, 37)	25.9	45	9.88
2	body weight (g)	135	106.13	15.69	106.46 (94.14, 115.84)	76.84	150.32	14.78
	shank length (cm)	135	3.08	0.43	3.06 (2.7, 3.5)	2.3	3.85	14.05
4	body weight (g)	137	218.63	37.28	214 (195, 243.86)	130	309	17.05
	shank length (cm)	137	4.3	0.43	4.3 (4, 4.6)	3.2	5.44	10.09
6	body weight (g)	167	414.93	61.85	407 (368.5, 463)	289	630	14.91
	shank length (cm)	167	5.78	0.48	5.78 (5.50, 6.10)	4.3	8.22	8.39
8	body weight (g)	174	621.29	91.28	610.5 (551.25, 688.75)	460	935	14.69
	shank length (cm)	174	6.83	0.5	6.8 (6.5, 7.2)	5.6	8.1	7.29
	body slope length (cm)	174	14.41	1	14.5 (13.73, 15)	11.3	17	6.92
	keel length (cm)	174	7.38	0.58	7.5 (7, 7.7)	6	9	7.87
12	body weight (g)	165	1111.78	174.56	1080 (986, 1227)	715	1675	15.70
	body slope length (cm)	165	18.24	1.38	18.2 (17.3, 19)	10.65	22	7.58
	keel length(cm)	165	9.53	0.76	9.5 (9, 10)	6.2	11.6	7.97
	chest width (cm)	165	6.26	0.64	6.3 (5.8, 6.8)	4.7	7.8	10.22
	chest depth (cm)	165	10.23	0.85	10.3 (9.8, 10.8)	7.5	12.2	8.32
	shank length (cm)	165	9.01	0.66	9 (8.5, 9.5)	7.4	10.3	7.29
	shank girth (cm)	165	3.79	0.3	3.8 (3.5, 4)	3	4.8	8.04
	pelvic width (cm)	165	2.38	0.24	2.4 (2.3, 2.6)	1.6	3	10.29

Note: P25 and P75 indicate the 25th and 75th percentiles, respectively. CV represents the coefficient of variation.

**Table 2:** Statistical result of carcass traits in Yanying chickens.

Traits	N	Mean	SD	Median (P25, P75)	Min	Max	CV (%)
dressed weight (g)	192	1018.88	163.02	995 (892, 1128)	713	1561	16.00
breast muscle weight (g)	192	111.95	25.73	110.8 (94.3, 127.95)	52.4	199.4	22.98
heart weight (g)	189	6.67	1.36	6.5 (5.6, 7.6)	4.2	10.7	20.47
gizzard weight (g)	188	20.31	3.96	19.7 (17.8, 22.43)	12.3	34.1	19.49
glandular stomach weight (g)	186	4.85	1.19	4.6 (4, 5.5)	2.5	8.7	24.65
spleen weight (g)	188	3.39	1.09	3.15 (2.6, 4.1)	1.6	7.5	32.10
liver weight (g)	189	33.71	8.48	32.2 (28.3, 37.8)	4.46	66.4	25.16
lung weight (g)	188	9.57	2.49	9 (7.5, 11.3)	5.6	16.8	26.06
leg muscle weight (g)	190	162.82	34.92	156.6 (139.8, 183.35)	97.4	271.8	21.45
skin fat weight (g)	192	65.78	14.7	64.2 (56.6, 74.93)	8.14	110.1	22.35
foot weight (g)	192	55.14	12.44	52.7 (44.95, 64.6)	34	85	22.56
head weight (g)	190	41.03	7.84	39.05 (35.23, 45.4)	25.9	66.4	19.11
half-eviscerated weight with giblets (g)	192	902.83	152.49	884.75 (790.95, 997.4)	613.4	1429.8	16.89
giblets weight (g)	191	79.07	12.88	78.1 (70.4, 86.25)	40.6	127	16.28
eviscerated weight (g)	192	824.2	145.24	807.3 (720.35, 904.98)	514.2	1325.6	17.62
half-eviscerated yield with giblets (%)	186	79.00	3.59	79.12 (77.59, 80.71)	47.24	86.64	4.54
breast muscle yield (%)	192	13.55	1.84	13.64 (12.31, 15.03)	5.94	17.68	13.56
leg muscle yield (%)	190	19.66	1.65	19.74 (18.65, 20.85)	13.61	23.34	8.40
breast muscle thickness (mm)	190	12.63	2.59	12.73 (10.9, 14.43)	5.16	18.77	20.52

Note: P25 and P75 indicate the 25th and 75th percentiles, respectively. CV represents the coefficient of variatio.

**Table 3:** Meat quality trait statistics in Yanying chickens.

Traits	N	Mean	SD	Median (P25, P75)	Min	Max	CV (%)
pH	192	5.69	0.79	5.963 (5.17, 6.25)	3.52	7.11	13.82
L*	192	59.54	6.32	59.85 (56.2, 63.7)	35.02	80.19	10.61
a*	192	6.69	3.35	6.13 (4.45, 8.29)	1	19.47	50.06
b*	192	7.79	2.92	7.86 (5.41, 9.93)	0.67	16.5	37.44
Shear force (N)	184	44.18	17.16	40.23 (31.54, 52.2)	15.85	104.83	38.84
intramuscular fat of dried meat (%)	179	27.84	9.15	27.2 (21.78, 33.41)	10.82	71.13	32.88
intramuscular fat of fresh meat (%)	179	2.61	0.79	2.55 (2.07, 3.07)	0.99	5.42	30.19
moisture content (%)	184	70.84	1.58	70.89 (70.08, 71.69)	62.24	75.30	2.22
fat content (%)	183	3.34	1.35	3.24 (2.64, 4.08)	0.065	13.97	40.47
protein content (%)	184	22.99	2.26	23.05 (21.36, 24.71)	15.90	30.36	9.84

Note: P25 and P75 indicate the 25th and 75th percentiles, respectively. CV represents the coefficient of variation.

Sex effects were further evaluated for each of the three trait groups. Among the 19 growth traits, no significant sex differences were detected from 0 to 4 wk. Significant differences (*P* < 0.05) in shank length first appeared at 6 wk. From 8 to 12 wk, all growth traits except pelvic width showed significant sex differences (*P* < 0.05). Extremely significant differences (*P* < 0.0001) were detected for body weight at 8 and 12 wk, and at 12 wk for body slope length, keel length, chest depth, shank length, and shank girth (Fig. 1). Among the 29 carcass and meat quality traits, 15 showed significant sex differences (*P* < 0.05), including 12 traits with highly significant differences (*P* < 0.0001). These traits included dressed weight, foot weight, heart weight, liver weight, lung weight, leg muscle weight, skin fat weight, head weight, giblet weight, eviscerated weight, and breast muscle yield (Fig. 2). Based on these results, sex was included as a covariate in subsequent analyses.

**Fig. 1:**
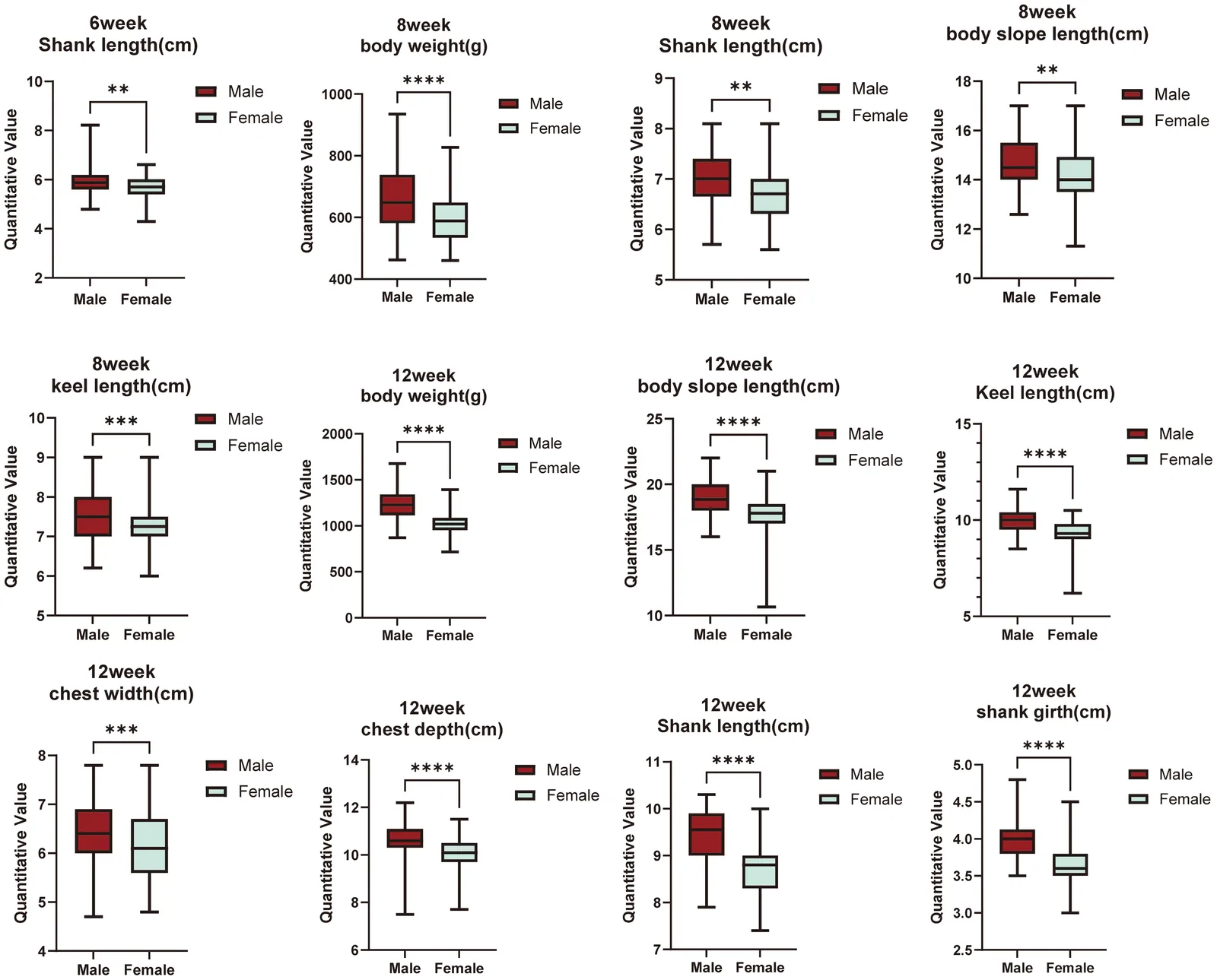
Comparison of growth traits between male and female Yanying chickens. The y-axis represents the quantitative value of each trait. ** represents *P* < 0.01, *** represents *P* < 0.001 and **** represents *P* < 0.0001.

**Fig. 2:**
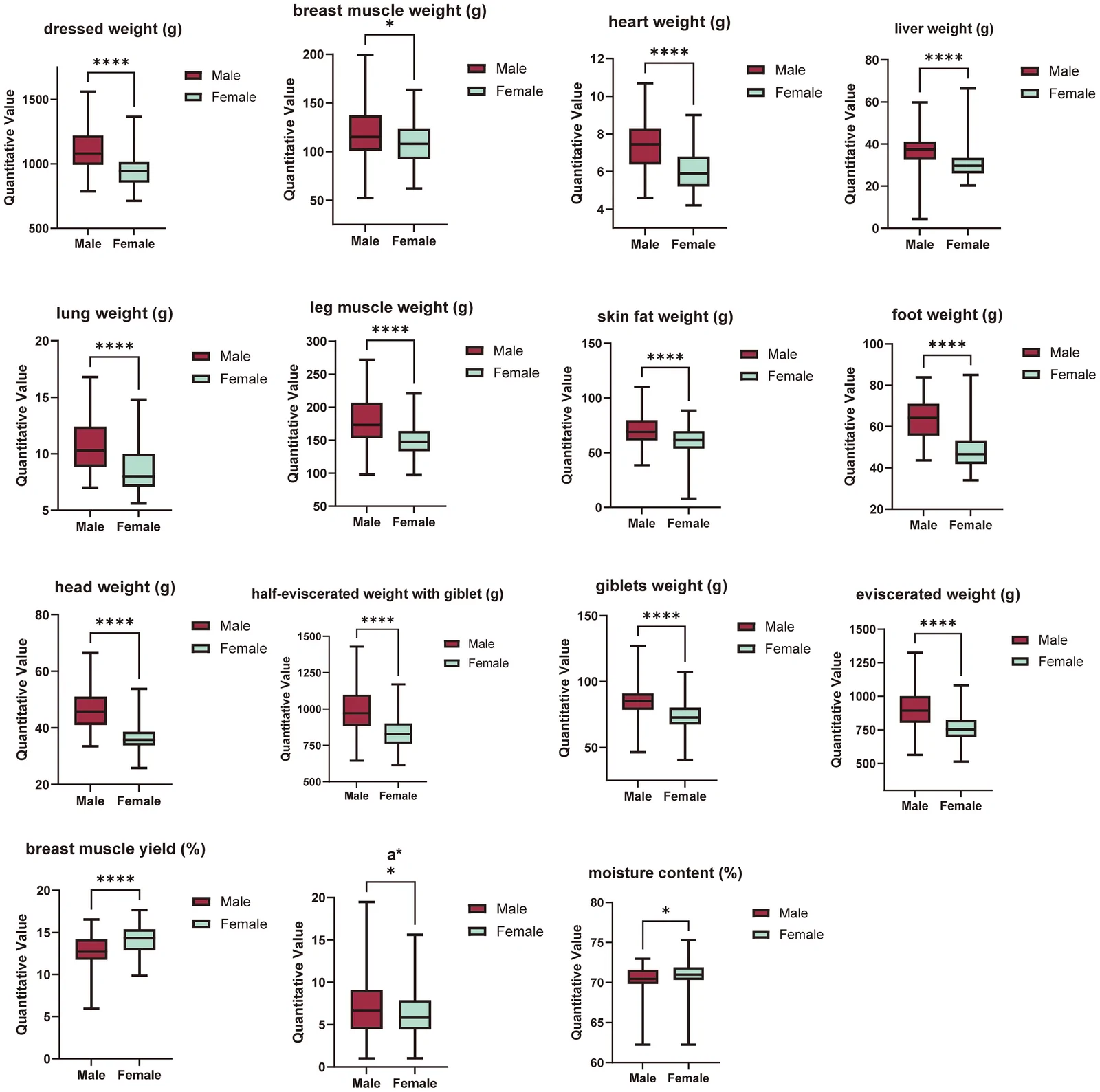
Comparison of carcass and meat quality traits between male and female Yanying chickens. The y-axis represents quantitative value of each trait. * represents *P* < 0.05 and **** represents *P* < 0.0001.

### GWAS Identifies candidate genes for growth, carcass and meat quality traits

Based on whole-genome resequencing data, PCA indicated limited population structure among Yanying chickens (Supplementary Fig. S3). Population structure and relatedness were modeled using a genome-wide kinship matrix, without additional PC covariates. GWAS was then performed for 19 growth traits and 29 carcass and meat quality traits. For each trait, the effective sample size used in the GWAS corresponded to the number of individuals with valid phenotype records reported in the Phenotypic Statistics section (Supplementary Tables S5 and S6). Using the stringent threshold of -log_10_*P* ≥ 9.07, a total of 455 significant SNPs were identified (Fig. 3A-C; Supplementary Table S1). Among them, 453 SNPs were associated with carcass and meat quality traits, whereas 2 SNPs were associated with growth traits. Annotation of the 455 significant SNPs showed that 130 SNPs were located in intergenic regions, 228 in intronic regions, 49 upstream of coding sequences, and 31 downstream of coding sequences, with the remaining SNPs assigned to other annotation categories (Supplementary Table S1). In total, 176 candidate genes were annotated for carcass and meat quality traits, whereas no candidate gene was annotated for growth traits. Representative significant SNPs and their corresponding annotated candidate genes are shown in Table 4, and the complete list of significant SNPs and annotated genes is provided in Supplementary Table S1. Representative candidate genes included *FTO* for liver weight, *ERBB4* and *ROBO2* for head weight, *MAP3K12* and *CDH6* for breast muscle thickness, *ARNTL* for breast muscle pH, *EFR3A* for intramuscular fat related traits, and *AGRN* for fat content.

**Fig. 3:**
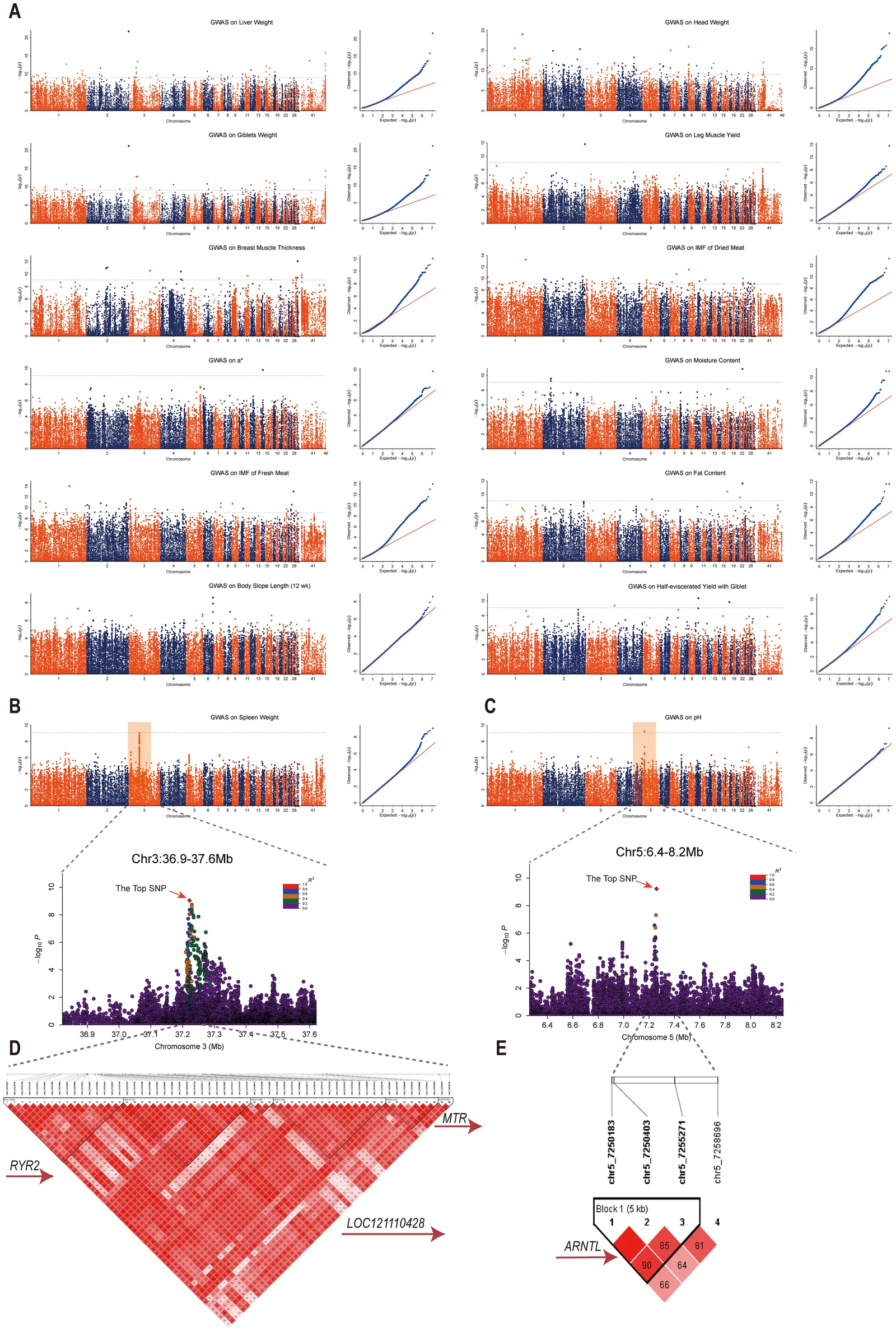
Manhattan plots, Q-Q plots, regional association plots, and LD analyses for selected growth, carcass, and meat quality traits. (A) Manhattan plots and corresponding Q-Q plots for traits with significant GWAS signals. The horizontal dashed line indicates the Bonferroni-corrected significance threshold (-log_10_*P* = 9.07). (B) Manhattan plot and regional association plot for spleen weight. The highlighted candidate region is located on chromosome 3 (36.9-37.6 Mb), and the top SNP is indicated. (C) Manhattan plot and regional association plot for breast muscle pH. The highlighted candidate region is located on chromosome 5 (6.4-8.2 Mb), and the top SNP is indicated. (D) LD analysis of SNPs with r^2^ > 0.4 relative to the top SNP for spleen weight on chromosome 3. Candidate genes in this region include *RYR2, LOC121110428*, and *MTR*. (E) LD analysis of SNPs with r^2^ > 0.4 relative to the top SNP for breast muscle pH on chromosome 5. The associated LD block is located upstream of *ARNTL*.

**Table 4:** Representative significant GWAS SNPs and annotated genes.

Traits	Chr	SNP	-log_10_*P*	REF	ALT	Gene
liver weight	2	148829507	21.67527462	T	G	*LOC121113108*
	41	84959344	15.84029013	A	G	*SRFBP1*
	41	28513672	13.66396794	A	T	*LOC107052258*
	3	30813067	13.42696138	C	T	*LTBP1*
	41	84956997	12.81595836	A	G	*SRFBP1*
head weight	1	124549586	19.00847853	T	C	*LOC768434*
	9	1708587	15.90563589	G	C	*/*
	1	97198865	15.56216161	C	A	*ROBO2*
	2	131532599	15.31501507	C	T	*NUDCD1*
	7	3547628	15.02006494	C	T	*ERBB4*
giblets weight	2	148829507	21.15740159	T	G	*LOC121113108*
	41	84959344	14.32054338	A	G	*SRFBP1*
	3	30813067	12.80935683	C	T	*LTBP1*
	3	25896433	12.75225606	A	G	*SRBD1*
	41	84956997	12.70630718	A	G	*SRFBP1*
leg muscle yield	2	148820080	11.81011807	T	G	*LOC121112883*
half-eviscerated yield with giblets	10	10430911	10.3750243	T	A	LOC124417112
	18	222461	9.8541573	T	C	LOC121107158
	3	101887693	9.365734908	C	T	/
breast muscle thickness	34	1024598	12.09111339	C	T	*MAP3K12*
	2	73361930	11.07630401	G	A	*/*
	2	69114653	10.9517129	G	A	*CDH6*
	3	74449219	10.56924439	A	C	*/*
	4	72795583	10.41734331	A	G	*RBPJ*
pH	5	7258696	9.220104924	G	A	*ARNTL*
a*	14	7762066	9.791196516	G	A	*/*
IMF of dried meat	1	136436716	13.29550866	A	G	*LOC776067*
	9	2735475	11.56107378	A	G	*GPC2*
	7	24604875	10.78994761	G	A	*CNTNAP5*
	2	140306035	10.36831613	T	C	*EFR3A*
	1	1853606	10.27887417	A	T	*LOC416696*
IMF of fresh meat	1	136436716	14.02138034	A	G	*LOC776067*
	28	173396	13.02754621	A	C	*/*
	3	5531630	11.58095271	A	G	*MACROD2*
	1	32403523	11.16499815	T	A	*/*
	2	140306035	10.87195536	T	C	*EFR3A*
moisture content	22	4673649	10.91080512	C	T	*/*
	22	4673651	10.91080512	G	C	*/*
	2	28408610	9.596826572	G	A	*BZW2*
	2	28408752	9.563985728	G	A	*BZW2*
	2	28408771	9.563985728	A	G	*BZW2*
fat content	22	4673649	11.61573706	C	T	*/*
	22	4673651	11.61573706	G	C	*/*
	17	5238019	10.42349791	G	C	*CDK5RAP2*
	21	2756958	9.531495126	T	C	*AGRN*
	5	33561421	9.255971407	T	G	*LOC101747910*
body slope length (12 wk)	6	35528301	9.916940925	G	A	*/*
	6	35528924	9.138736892	C	T	*/*

Note: For each trait group, only the top five significant SNPs ranked by -log_10_*P* are shown. “/” indicates that no annotated candidate gene was identified for the corresponding SNP within the annotation region.

### LD Analysis for spleen weight and breast muscle pH

Based on the GWAS results, spleen weight and breast muscle pH showed distinct regional association patterns on chromosomes 3 and 5, respectively, characterized by clusters of neighboring associated SNPs in the Manhattan plots and deviations from the expected distribution at higher -log_10_*P* values in the corresponding Q-Q plots. However, we observed that the top SNP for spleen weight (chr3:37221717; -log_10_*P* = 9.04) fell slightly below the stringent threshold of 9.07 but exceeded the Bonferroni-corrected genome-wide significance threshold of 8.38 (Supplementary Table S2). To further refine these loci, LocusZoom analysis was performed around the top SNP (± 1 Mb) for spleen weight (chr3:37221717) and the top SNP for breast muscle pH (chr5:7258696). SNPs with r^2^ > 0.4 were retained for subsequent LD block analysis and gene annotation.

For spleen weight, six LD blocks were identified in the candidate region (Fig. 3D, see Supplementary Table S2 for details). These blocks contained or were located near the candidate genes *RYR2, LOC121110428*, and *MTR*. The top SNP chr3:37221717 was located within Block 4, which was annotated to *LOC121110428*. Physically, this lead variant was 8610-11365 bp away from variants within *RYR2*-containing Block 1, and showed moderate pairwise linkage disequilibrium (r^2^ = 0.52-0.59) with those variants. Block 1 included both *RYR2* and *LOC121110428*, and the variant associated with *RYR2* was located upstream of the coding region. Blocks 2 to 5 were all mapped to *LOC121110428*, whereas Block 6 was mapped to *MTR* and also represented an upstream variant.

For breast muscle pH, four highly correlated SNPs (r^2^ > 0.4) were retained, defining a single LD block on chromosome 5 (Fig. 3E, see Supplementary Table S2 for details). This block was located upstream of *ARNTL*. To further evaluate this candidate gene, a transcriptome-wide expression-trait association analysis was performed using breast muscle RNA-seq data. *ARNTL* expression exceeded the Bonferroni-corrected significance threshold (-log_10_*P* ≥ 5.71) in the Manhattan plot (Fig. 4), which was consistent with the GWAS signal.

**Fig. 4:**
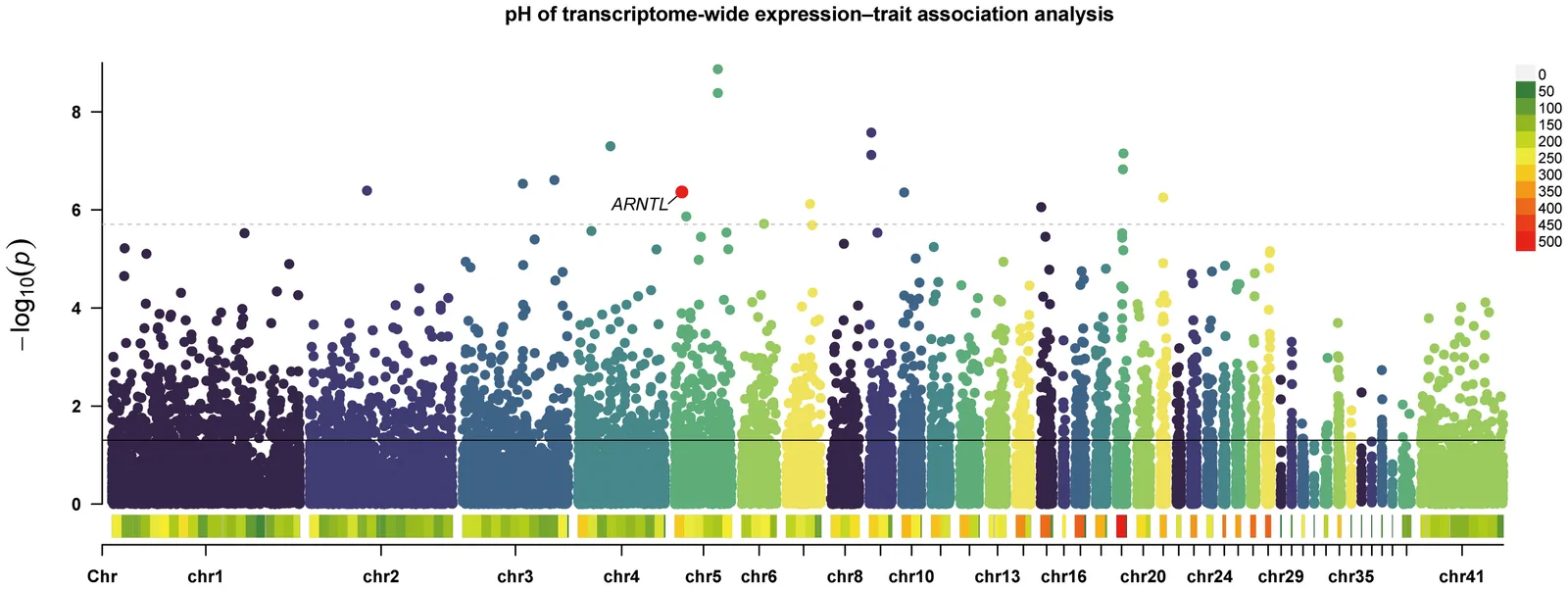
Manhattan plot of the transcriptome-wide expression-trait association analysis for muscle pH. Each dot represents the -log_10_*P* value for a gene, plotted by genomic position. The dashed line indicates the significance threshold (-log_10_*P* = 5.71). Colors reflect chromosomal density distribution. The *ARNTL* gene is highlighted in red, showing a significant association with muscle pH.

### Functional enrichment analysis of candidate genes

To further characterize the biological functions of candidate genes associated with carcass and meat quality traits, GO and KEGG enrichment analyses were performed for the 176 identified candidate genes. Using an adjusted *P*-value < 0.05 as the significance threshold, GO enrichment analysis identified 20 significantly enriched terms (Fig. 5A, see Supplementary Table S3 for details), including 10 **biological process (BP)** terms, 6 **cellular component (CC)** terms, and 4 **molecular function (MF)** terms. Among these, homophilic cell adhesion via plasma membrane adhesion molecules (GO:0007156) was among the most significantly enriched BP terms, whereas integral component of plasma membrane (GO:0005887) were prominent CC terms. In the MF category, calcium ion binding (GO:0005509) showed strong enrichment.

**Fig. 5:**
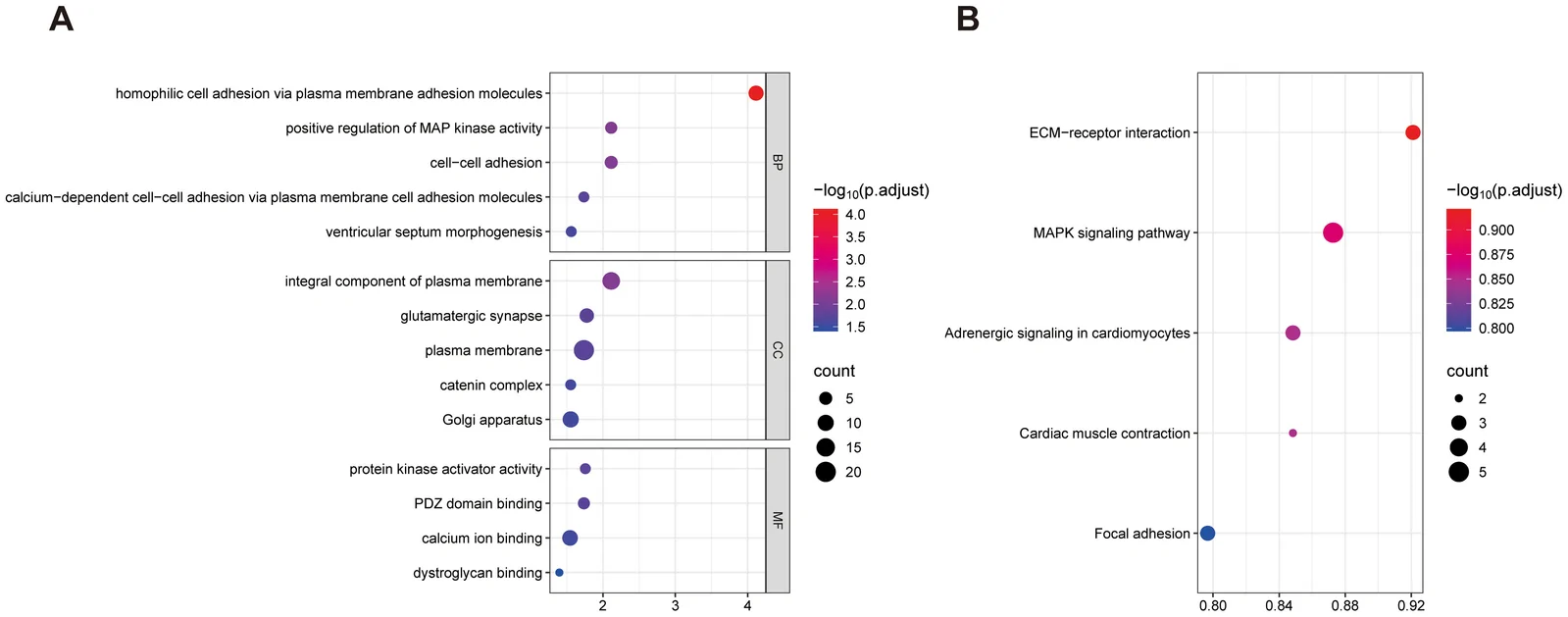
GO and KEGG enrichment analyses of candidate genes. (A) GO enrichment analysis. Enriched GO terms are shown for **biological process (BP), cellular component (CC)**, and **molecular function (MF)**. Dot size represents gene count, and dot color represents -log_10_ (adjusted *P*-value). Only the top five enriched GO terms in each category are shown. (B) KEGG enrichment analysis. Dot size represents gene count, and dot color represents −log_10_ (adjusted *P*-value). Only the top five KEGG pathways are shown.

KEGG enrichment analysis did not identify any pathways that remained significant after multiple-testing correction (adjusted *P* < 0.05). However, several pathways showed nominal enrichment based on raw *P* values (*P* < 0.05), including ECM-receptor interaction (gga04512), MAPK signaling pathway (gga04010), adrenergic signaling in cardiomyocytes (gga04261), and cardiac muscle contraction (gga04260) (Fig. 5B; Supplementary Table S4). These pathways were therefore considered suggestive and were used only for exploratory biological interpretation.

## Discussion

The growth, carcass and meat quality traits of poultry directly impact economic efficiency, meat quality and flavor, as well as consumer demand, making them key determinants in poultry farming and breeding.

In the present study, a broad set of growth, carcass, and meat quality traits was systematically characterized in Yanying chickens. Growth traits showed moderate phenotypic variation, with CV values ranging from 6.92% to 17.05%, whereas several carcass and meat quality traits exhibited greater dispersion. Notably, a* (50.06%) and b* (37.44%) showed relatively high CV values, indicating substantial individual variation in meat color. However, no clear candidate genes were identified for these traits in the subsequent analysis. As meat color is a complex quantitative trait, its high phenotypic dispersion may be influenced by postmortem metabolic processes, bleed-out efficiency and other environmental factors (Alvarado et al., 2007; Ramanathan et al., 2020), while the absence of clear candidate genes does not exclude a polygenic genetic basis. Overall, the Yanying chicken population retains appreciable phenotypic diversity for economically important traits, providing a useful basis for genetic association analysis. In addition, sex differences in growth traits emerged at 6 wk and became more pronounced by 8-12 wk, while significant sex effects were also observed for several carcass and meat quality traits at 12 wk. In particular, sex-related differences appeared later in Yanying chickens than in commercial broilers (Livingston et al., 2020), which may reflect a breed-specific growth pattern. Accordingly, sex was included as a covariate in the GWAS model to reduce potential confounding effects. Based on the observed phenotypic variation and the quantitative distribution of most traits, this population was suitable for subsequent analysis.

A preliminary comparison of the 176 candidate genes with previous studies showed that only 1 gene-trait association had been directly reported for the corresponding traits in chickens. Approximately 5 others had been indirectly linked to related traits in poultry. Thus, approximately 170 candidate genes remain relatively unexplored for the traits investigated here (Supplementary Table S1). For instance, *FTO* polymorphisms have been reported to be significantly associated with carcass traits in chickens, including liver weight, head weight, heart weight, and leg muscle weight (Ding et al., 2025). In the present study, *FTO* was also identified in association with liver weight, indicating a potential role in carcass trait variation. *AGRN* has been reported as a key hub gene in a module related to adipose tissue growth and development in chickens (Gao et al., 2020), and its association with fat content in the present study suggests that it may also be involved in lipid deposition. In addition, we also found that *EFR3A* serves as a candidate gene for IMF of dried meat in our study. Although *EFR3A* has been mapped to a QTL region related to fat deposition in cattle (Leonard and Pausch, 2022), evidence in chickens remains limited. Therefore, *EFR3A* may represent a candidate gene that warrants further investigation in chicken fat related traits. Overall, these results indicate that some of the loci identified in this study overlap with previously reported genes, whereas others may represent potentially novel candidates requiring further validation.

Not all significant SNPs identified in this study overlapped with previously reported loci, further suggesting the genetic complexity of meat quality traits in chickens. Breast muscle pH is an important meat quality trait because it affects flavor, tenderness, and shelf life (Mounier et al., 2006). In other chicken populations, *PPP1R3A* (Le Bihan-Duval et al., 2018), *PITX2* (Cao et al., 2021), and *GALNT* (Munyaneza et al., 2022) have been proposed as candidate genes associated with pH related traits. In contrast, *ARNTL* emerged as a potential candidate gene for breast muscle pH in the present Yanying chicken population, suggesting that the genetic basis of this trait may differ among chicken populations. *ARNTL*, also known as *BMAL1*, is a core circadian regulator and has been implicated in muscle metabolism and glucose utilization (Harfmann et al., 2016; Takahashi, 2017; Sato et al., 2018). Previous studies have also reported crosstalk between BMAL1 and hypoxia related signaling pathways (Peek et al., 2017; Wu et al., 2017; Zhang et al., 2020), which offers a partial biological rationale for further investigation of *ARNTL* in high altitude chickens. Taken together, these observations provide a biological rationale for considering *ARNTL* as a plausible candidate gene for breast muscle pH, while further functional validation is required to clarify its role in chickens.

In addition to the *ARNTL* locus associated with breast muscle pH, GWAS also identified a distinct candidate region for spleen weight on chromosome 3. The top SNP for spleen weight was located at chr3:37221717, within a region containing *LOC121110428* and *RYR2. RYR2* encodes a major Ca²⁺ release channel involved in intracellular calcium signaling (Laitinen et al., 2001). Although there is currently no direct evidence linking *RYR2* to spleen weight in chickens, previous mouse studies have shown that abnormal RyR2 function can be accompanied by increased thymus and spleen weights (Nishio et al., 2006, 2008, 2014), suggesting a possible relationship with lymphoid organ phenotypes. However, these observations were derived from a pathological R420W knock-in mouse model and therefore provide only indirect evidence for the physiological regulation of spleen development. In addition, hypoxia has been reported to affect spleen related immune traits in chickens (Wang et al., 2019), which may be relevant when interpreting this signal in a high altitude population. Recent population genomic studies in Tibetan chickens also identified *RYR2* as a candidate gene under selection (Li et al., 2023), providing only indirect evidence for its possible relevance in high-altitude chickens. Taken together, these findings point to *RYR2* as one positional candidate gene for spleen weight in Yanying chickens, but do not exclude a contribution from *LOC121110428*. Further functional validation is required to clarify their roles in spleen weight variation.

Functional enrichment analysis of the 176 candidate genes associated with carcass and meat quality traits identified significant enrichment of GO terms related to homophilic cell adhesion via plasma membrane adhesion molecules (GO:0007156) and calcium ion binding (GO:0005509), whereas ECM-receptor interaction (gga04512) and the MAPK signaling pathway (gga04010) showed only nominal enrichment in the KEGG analysis. These enriched terms and pathways suggest that cell adhesion, calcium related functions, and extracellular matrix associated signaling may be involved in the BP underlying carcass and meat quality traits in Yanying chickens. Previous studies have shown that cell adhesion and extracellular matrix interactions are closely related to muscle structure and tissue properties (Engler et al., 2004; Gokgol et al., 2012; Mertz et al., 2013), whereas calcium related pathways play important roles in muscle development, myofiber organization, and intracellular signaling (Marciano et al., 2021; Mo et al., 2023). In addition, ECM-receptor interaction and MAPK signaling have previously been associated with muscle fiber integrity and intramuscular fat deposition in poultry (Cui et al., 2012). Taken together, these results suggest that cell adhesion, calcium related functions, and extracellular matrix associated pathways may be involved in carcass and meat quality traits in Yanying chickens.

Some limitations should be considered when interpreting these results. The sample size was relatively limited, and the number of animals included varied among traits, which may have reduced the power to detect loci with small effects. In addition, all animals were derived from a single Yanying chicken population, and the associations identified in this study therefore need to be further examined in independent populations. Although the regional association, LD, and expression-based analyses provided additional evidence for several candidate loci, these results do not establish a causal role for the candidate genes, and further functional validation is still needed.

## Conclusion

In summary, this study systematically evaluated growth, carcass, and meat quality traits in Yanying chickens and identified substantial phenotypic variation within the population. GWAS detected 455 significant SNPs associated with these traits and identified 176 candidate genes, suggesting a complex genetic basis underlying economically important traits in this indigenous breed. Some of the identified loci overlapped with previously reported genes, whereas others may represent candidate loci of particular interest in Yanying chickens. Among them, *ARNTL* and *RYR2* were highlighted as positional candidate genes associated with breast muscle pH and spleen weight, respectively, with expression-based analysis providing additional evidence for *ARNTL*. Functional enrichment analysis suggested that cell adhesion, calcium related functions, extracellular matrix interaction, and nominally enriched MAPK signaling pathways may be involved in carcass and meat quality traits. Overall, these findings improve our understanding of the genetic basis of growth, carcass, and meat quality traits in Yanying chickens and provide candidate loci and genes for future functional validation and genetic improvement of indigenous chicken breeds.

## Author contributions

Yuhang He performed the genomic and statistical analyses, interpreted the results, and drafted the manuscript. Qiang Wang, Jianfeng Huang, and Renbo Tong organized and conducted the animal experiment and participated in phenotypic measurements and biological sample collection. Jianian Zhao, Jianxing Jiang and Xiyu Tan conducted the experiment in Xichang, including sample collection, slaughter procedures, and carcass phenotyping. Ruxue Liu, Qiao Xiang and Xinyu Wei performed growth-trait measurements at different developmental stages. Xiaochan Yuan and Zhaoyou Zheng, performed meat-quality measurements. Ming Yang and Yuqi Chen performed the linkage disequilibrium analyses. Jinghe Li and Jun Zou conducted SNP annotation and GO and KEGG enrichment analyses. Hehe Liu conceived and designed the study and supervised its implementation. Anqi Huang participated in the analysis and interpretation of the results and co-drafted the manuscript. All authors read and approved the final manuscript and agreed to be accountable for their respective contributions.

## Data availability

The raw sequence data reported in this paper have been deposited in the Genome Sequence Archive (Genomics, Proteomics & Bioinformatics 2025) in National Genomics Data Center (Nucleic Acids Res 2025), China National Center for Bioinformation / Beijing Institute of Genomics, Chinese Academy of Sciences (GSA: CRA048833) that are publicly accessible at https://ngdc.cncb.ac.cn/gsa.

## Disclosures

The authors declare that they have no conflicts of interest related to this study.

## Ethics declaration

This study was conducted in accordance with the ARRIVE (Animal Research: Reporting of In Vivo Experiments) guidelines as well as the following guidelines for animal welfare and/or reporting: Guide for the Care and Use of Agricultural Animals in Research and Teaching, 4th edition, 2020. This study was approved by the Animal Welfare Committee of Sichuan Agricultural University. (Approval No. 20230169)

## Footnotes

Supplementary material associated with this article can be found, in the online version, at doi:10.1016/j.psj.2026.107768.
