# Supplementary material for: Genome-wide association study of growth, carcass, and meat quality traits in Liangshan Yanying chickens
Source: Poult Sci. 2026 Sep 12;105(12):107768. doi: 10.1016/j.psj.2026.107768 (PMC13631890; doi:10.1016/j.psj.2026.107768)
Supplement: Supplementary file 1 [file mmc1.docx]

**Supplementary Figure S1.** Frequency distribution of growth traits. The X-axis shows trait values, and the Y-axis shows the number of values.
